# Substitution Rtq267h of Hepatitis B Virus Increases the Weight of Replication and Lamivudine Resistance

**DOI:** 10.5812/hepatmon.12160

**Published:** 2013-10-01

**Authors:** Bo Qin, Bo Zhang, Xiaodong Zhang, Tingting He, Wenying Xu, Lijun Fu, Chunyu Tu

**Affiliations:** 1 Shaoxing Centre for Disease Control and Prevention, Shaoxing, China; 2 State Key Lab of Virology, Wuhan Institute of Virology, Chinese Academy of Sciences, Wuhan, China; 3 College of Life Science, Shaoxing University, Shaoxing, China

**Keywords:** Hepatitis B Virus, Susceptibility, Mutation

## Abstract

**Background:**

Nucleus(t)ide analogs (NAs), containing Lamivudine (LMV), adefovir dipivoxil (ADV), endeavor (ETV), telbivudine (LdT), and tenofovir (TDF) are widely used for the treatment of chronic hepatitis B (CHB), but long term anti-Hepatitis B virus (HBV) therapy with NAs may give rise to the emergence of drug-resistant viral mutants.

**Objectives:**

This study aimed to find and identify some new resistance mutations of HBV from the patients accepted anti-HBV therapy.

**Patients and Methods:**

The reverse transcriptase (RT) coding region of HBV was PCR-amplified using HBV DNA extracted from patients' blood samples and sequenced.

**Results:**

Nineteen substitution mutations were detected. Among them, rtQ267H was often observed in patients receiving LMV administration. This LMV therapy-related mutation was introduced into HBV replication-competent plasmids. The in vitro susceptibility of both wild-type (WT) and mutant-type (MT) HBV to NAs was analyzed by Southern blot, and/or quantitative real-time PCR (qRT-PCR). The rtQ267H substitution enhanced HBV replication not merely in single-site mutation, but also in multisite mutations. The in vitro susceptibility analysis showed that the existence of rtQ267H in WT and LMV-resistant (LMVr) HBV were responsible for the reduced susceptibility to LMV to varying degrees, and enhanced HBV replication capacity. However, HBV harbored this substitution retained normal susceptibility to ADV, LdT, ETV, and TDF.

**Conclusions:**

The result suggested that rtQ267H is a potential adaptive mutation of HBV to LMV.

## 1. Background

Hepatitis B virus (HBV) belongs to hepadnavirus family and contains a 3.2 kb (1), relaxed circular (RC), partially double-stranded DNA genome with four overlapping open reading frames (ORFs): P, S, C, and X (2). All over the world, there are more than 350 million Chronic Hepatitis B (CHB) patients, and acute and chronic necroinflammatory liver diseases caused by HBV infection account for 0.5-1.2 million deaths each year (3). The replication of HBV is a complex and ingenious process, including one step of error-prone reverse transcription which yields an RNA intermediate, named pregenomic RNA (pgRNA), analogous to human immunodeficiency virus (HIV) (2). Just because of this, HBV exists in infected individuals as a quasispecies which is an equilibrium distribution of closely related gene sequences. HBV virions with selection advantage can be selected under different selection pressures, such as immunity of the organism, drug therapy, and so on (4). So virions carrying relevant resistance mutations survive reasonably after treatment with NA drugs. To date, complete HBV eradication from hepatocyte is impossible with currently available agents (5), containing interferon and NAs which are widely used for anti-HBV therapy (6). NAs inhibit HBV replication effectively in CHB patients and significantly reduce mortality, the risk of cirrhosis-related complications, the incidence of hepatocellular carcinoma (HCC), and so on (7). However, the frequent emergence of drug- resistant mutations often leads to anti-HBV treatment failure and liver disease progression (8).

HBV polymerase is divided into four domains: terminal protein, spacer, reverse transcriptase (RT), and RNase H (9, 10). A variety of NAs-resistant mutations of HBV have been discovered and confirmed, all of which were located in the RT, which is further subdivided into seven subdomains: A to G (8). Substitution rtA181V/T and/or rtN236T can reduce the anti-HBV effect of ADV, rtN238R, rtT240Y and rtN248H of HBV were newly reported to decrease susceptibility to ADV (8). A combination of mutations in the B, C, or D domain, such as rtI169T, rtM250I/V, and a background of rtM204V/I confer resistance to ETV (8). Substitution rtA194T was reported to be a TDF resistance mutation which needs further confirmation. Mutations rtP177G and rtF249A predicted by computer were reported lately to confer resistance to TDF both in vivo and in vitro (11). The most common resistance mutations to LMV and LdT are rtM204I/V, located at the YMDD motif within the C domain of RT, usually accompanied with compensatory mutations of rtL180M and/or rtV173L to restore HBV replication capacity (12).

## 2. Objectives

This study aimed to identify novel potential drug- resistant mutations through the analysis of HBV-RT variation from CHB patients. HBV sequences from ten CHB patients undergoing LMV therapies were analyzed, and many substitution mutations were found. Among them, rtQ267H was found to be closely related to LMV therapy. This newly discovered mutation was then introduced into wild-type (WT) and mutant (MT) HBV replication-competent plasmids. In vitro analysis demonstrated that rtQ267H, rtM204V/Q267H, rtL180M/M204V, and rtL180M/M204V/Q267H resulted in 1.41-, 24.96-, 27.43, and 30.76-fold increases in resistance toward LMV, and 1.86-, 7.28-, 7.17- and 8.93-fold increases in resistance toward LdT, respectively. Although rtQ267H only led to slight resistance to LMV and LdT, enhanced HBV replication, aggravated existing LMV-resistance, and was proved to be another adaptive mutation of LMV, except rtL180M and rtV173L. This study could broaden the drug-resistance spectrum of HBV, and provide certain specific references for clinical drug administration.

## 3. Patients and Methods

### 3.1. CHB Patients and HBV-RT Sequence Analysis

As shown in our previous study, five patients who received ADV/switch to ADV from LMV treatment were studied in our previous study which aimed to find ADV-related mutations (13). In the present study, ten patients who received LMV treatment were selected. When HBV breakthrough was taken place, which was indicated by HBV DNA copy number and the serum alanine aminotransferase (ALT) as the references, serum samples from the veins of CHB patients were collected, from which HBV DNA was isolated using the QIAamp DNA Blood Mini Kit (Qiagen, Hilden, Germany). The HBV-RT segment was amplified by PCR with the following primer pair: RT-F and RT-R (Table 1), and cloned into the pCR2.1 vector directly. Five clones of each PCR product were two-way sequenced, and aligned with WT HBV-RT sequence (GenBank accession no. X02763.1, genotype A2, subtype adw2). Mutations appeared in high frequency were focused, and the association with the use of LMV was studied.

### 3.2. Plasmid Constructs

HBV mutants (MTs) were constructed using fusion-PCR with Primer-Fusion and Primer-rtL180M, -rtM204V/I, -rtQ267H (Table 1) harboring aimed mutations and pBSK-HBV1.3 as template, which is a replication-competent plasmid containing 1.3-folds over-length HBV genome (GenBank accession no. X02763.1, subtype adw2, genotype A2), with pBluescript KS+ backbone ( 11 ). Amphimutation and triple mutations were acquired by digestion and connection with different single mutants. Plasmids containing mutated sites were identified by sequence analysis.

**Table 1.** Primer Sequences for the Construction of MT ^a ^HBV Plasmids and qRT-PCR ^a ^

Primer Name, Type	Sequence (5’→3’)	Amplicon size, bp
**Primer-HBV-RC**		
Sense	GTTGCCCGTTTGTCCTCTAATTC	100
Anti-sense	GGAGGGATACATAGAGGTTCCTT	
**Primer-HBV-RT**		
Sense	CTAGGACCCCTGCTCGTGTT	843
Anti-sense	CGCAAACCCCAAAAGACCCA	
**Primer-Fusion**		
Sense	TCTTCTCGAGGATTGGGGACC	1257
Anti-sense	GCAGCCATGGAAACGATGTAT	
**Primer- rtL180M**		
Sense	GTTTCTC*A*TGGCTCAGTTTACTAG	717
Anti-sense	CTAGTAAACTGAGCCA*T*GAGAAAC	561
**Primer-rtM204V/I**		
Sense ^b^	CTTTCAGTTAT***G***(***C***)TGGATGATGTGGT	653
Anti-sense ^b^	ACCACATCATCCA***C***(***G***)ATAACTGAAAG	630
**Primer- rtQ267H**		
Sense ^b^	CAAGAACACATCATA***CAT***AAAATCAAAG	486
Anti-sense ^b^	CTTTGATTTT***ATG***TATGATGTGTTCTTG	818

^a^ Abbreviations: MT, mutant; qRT-PCR, quantitative real-time PCR

^b^ bold text, italics, substitution mutation for mutant HBV plasmids construction

### 3.3. Nucleoside Analogues (NAs)

NAs including 2',3'-dideoxy-3'-thiacytidine (LMV; Glaxo Smith Kline, Brentford, Middlesex, The UK), ADV (Gilead Sciences, Foster City, CA, The USA), ETV (Bristol-Myers Squibb Co., New York, NY, The USA), LdT (Novartis Pharmaceuticals Canada Inc., Dorval, Quebec, Canada) and TDF (Gilead Sciences) were diluted according to the manufactures’ instructions and used at the indicated concentrations (14).

### 3.4. Cell Culture and Transfection

Hepatoma Cell Line Huh7 cells were cultured in the Dulbecco’s modified Eagle’s medium (DMEM; Invitrogen, Carlsbad, CA, The USA) at 37°C in a 5% CO2 atmosphere supplemented with 5-10% fetal bovine serum (FBS; Gibco, Carlsbad, CA, The USA), 2 mM of glutamine, 100 IU/mL of penicillin and 100 IU/mL of streptomycin. Huh7 cells were seeded in 6-well plates (1×106/well) and transfected with 3 μg WT/MTs HBV-bearing plasmid per well using lipofectamine 2000 (Invitrogen) with or without the indicated NA concentrations. For transfection experiments, replication-competent HBV plasmids were cotransfected with a SEAP expression vector into cells, the relative efficiency of transfection was estimated by detecting SEAP activity in the culture medium using a chemiluminescent detection method (14).

### 3.5. Enzyme-Linked Immunosorbent Assay (ELISA)

Huh7 cells were transfected with the indicated replication-competent HBV plasmids, hepatitis B surface antigen (HBsAg), and hepatitis B e antigen (HBeAg) in the supernatant at 96-hour post transfection (hpt) was measured using a commercial diagnostic kit (Shanghai Kehua Diagnostic Medical Products Co. Ltd, Shanghai, China) according to the manufacturer’s instructions (14). The absorbance of controls and test specimens was determined with a precision spectrophotometer (BIO-RAD, iMARK 680, The USA) at 450/630 nm (450 nm reading wavelength with 630 nm reference wavelength. All samples from cultured supernatant were analyzed in triplicate.

### 3.6. Dry Chemical Scrip Assay

ALT of whole blood was detected by ALT scrip and Reflotron Plus instrument (Roche, Germany) according to the manufacturer’s instructions (15).

### 3.7. Detection of Encapsulated HBV DNA With Southern Blot

Huh7 cells (1 × 106/well in 6-well plate) were transfected with 3 μg of the plasmids pHBV1.3 and pHBV1.3-rtXs by using lipofectamine 2000 (Invitrogen), and cultured with or without the presence of different NAs at the indicated concentrations (11). Total HBV DNA replicative intermediates from intracellular core particles were extracted and subjected to agarose gel electrophoresis, followed by denaturation and Southern blotting with a 32P-labeled full length HBV probe according to previously published protocols (16, 17). Hybridization signals were visualized and analyzed using a phosphoimager (Cyclone, Packard Instrument Co., Inc., Meriden, CT, The USA). Data was quantified with OptiQuant software (Packard Bioscience Co., Inc., Meriden, CT, The USA).

### 3.8. Quantitative Real-Time PCR (qRT-PCR)

Cell lysis of Huh7 was treated with DNase I (Roche) at 37℃, 30 min to digest input plasmid DNA (11). Total HBV DNA replicative intermediates were purified from lysates of Huh7 cells 96 hpt and diluted in 20 μL water (containing RNase to digest yeast tRNA, DNase free), 2 μL of which was used as a template for qRT-PCR, which was conducted using SYBR Green I nucleic acid stain (Roche Diagnostics GmbH, Mannheim, Germany) on a Light Cycler real-time thermal cycler (Applied Biosystems, Foster City, CA, The USA) according to the manufacturers’ instructions (11). Primers HBV-RC-F and HBV-RC-R (Table 1) hybridized to the HBV surface gene were designed to quantify HBV-DNA RC genomes (100-bp fragment) by qRT-PCR relative to an external plasmid DNA standard (18). This method was also used to detect HBV DNA copies in the serum samples to evaluate the infection status in CHB patients (19). All samples were analyzed in triplicate.

### 3.9. Statistical Analysis

The statistical analysis was performed using Graph Pad (Graph Pad Software). Differences in multiple comparisons were determined for statistical significance using the Student’s T-test. P < 0.05 was considered as statistically significant. Results were presented as Means ± SD.

## 4. Results

### 4.1. Substitution rtQ267H was Related With LMV Therapy from Sequence Analysis

**Figure 1.**
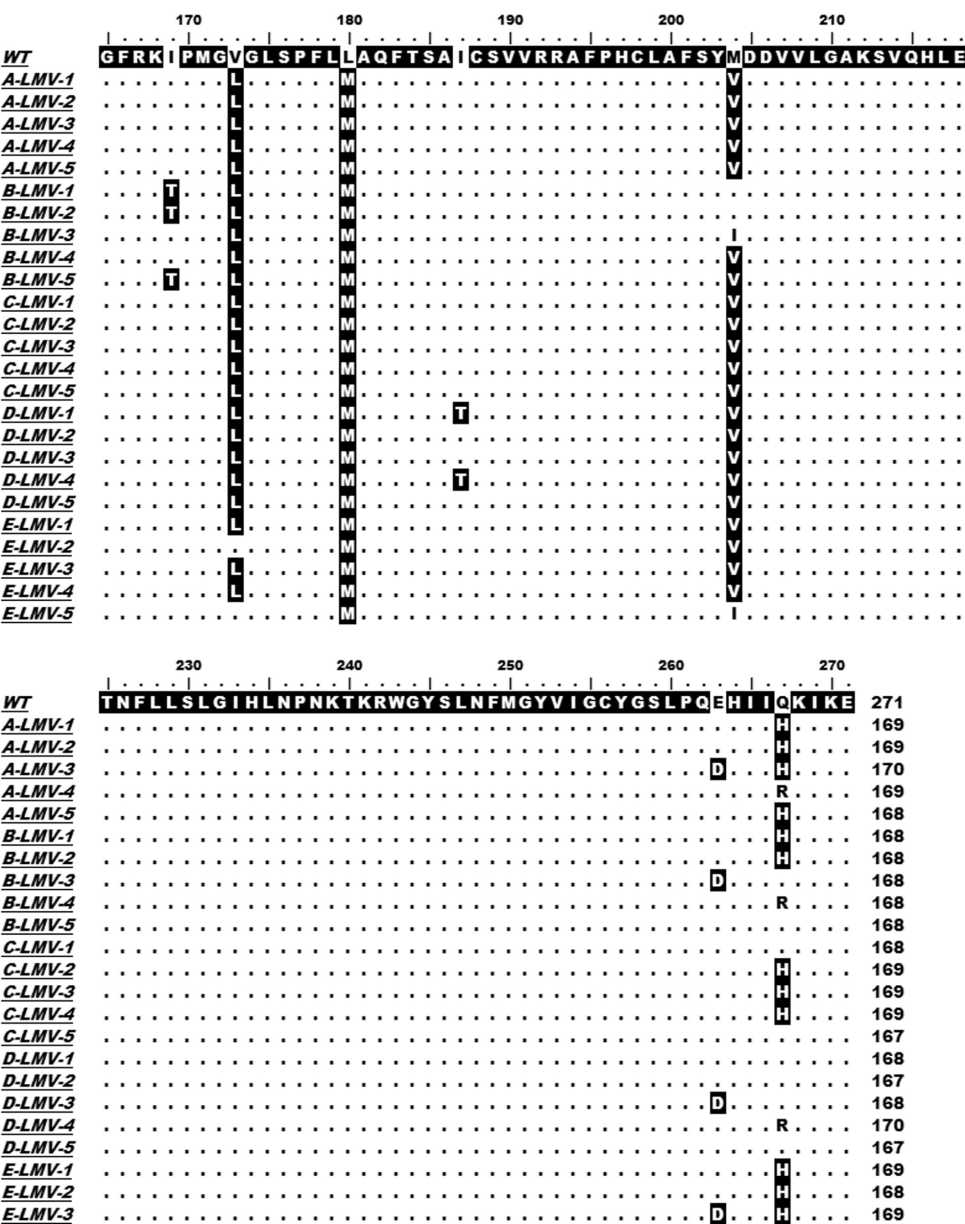
Sequence Alignments of HBV RT Regions HBV DNA was isolated from serum samples of five CHB patients (A, B, C, D and E) who treated with LMV. The HBV-RT segments were PCR-amplified and cloned. Five clones were sequenced for each sample. The sequence of each patient with LMV therapy was aligned with the reference sequence (WT, GenBank accession no. X02763.1, genotype A). The region (rt167 to rt270) contained the substitution sites are focused and shown.

Ten German CHB patients with a lengthy course (> 3 years) of disease which all took about two to three years LMV administration were taken into consideration in our present study. An obvious increase in ALT and/or HBV DNA levels might indicate HBV viral breakthrough and variation. The HBV-RT segment was amplified by PCR using HBV DNA extracted from the serum sample as template, RT-F and RT-R listed in Table 1 as primer pair, and two-way sequenced. All isolated HBV strains were A2 genotype. Sequence alignments with HBV-RT reference sequence indicated a variety of mutations in CHB patients after LMV and/or ADV treatment, which were reported in our previous study. After LMV treatment, interestingly a novel mutation, rtQ267H was found, except rtM204V and/or rtL180M emerged as described in previous reports, in all of the ten patients, and took place in five of them in high-frequency, as shown in Figure 1.

The relatively high prevalence of this substitution in CHB patients with LMV therapy implies that it might be selected after LMV long-term usage.

### 4.2. The Replication Capacity of MT pHBV1.3 in Vitro

**Figure 2.**
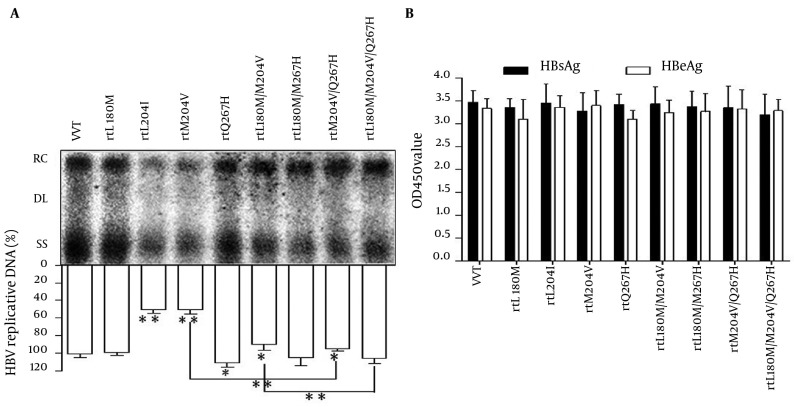
Replication Ability and Antigen Expression Levels of WT HBV/Mutants Huh7 cells were transfected with WT or MT HBV plasmids. (A) Encapsulated viral DNA was extracted and detected by Southern blot. Relaxed circular (RC), double stranded linear (DL) and single stranded (SS) HBV DNAs are demonstrated. The relative level of WT HBV replication capacity is shown as a percentage of the control by gray analysis (upper panel). (B) HBsAg and HBeAg in the supernatant were detected using an ELISA diagnostic kit (Shanghai Kehua Diagnostic Medical Products Co., Ltd.) according to the manufacturer’s instructions. Each value is the mean of at least 3 independent experiments. The error bars represent the standard deviation (SD). Statistically significant differences between the groups are displayed as * (P < 0.05) or ** (P < 0.01).

To further analyze the influence of the rtQ267H on HBV replication and sensitivity to LMV, MT HBV replication-competent plasmids, pHBV-rtQ267H, -rtL180M, -rtM204V, -rtM204I, -rtL180M/M204V, -rtL180M/Q267H, -rtM204V/Q267H, -rtL180M/M204V/Q267H were constructed based on pHBV1.3. MT HBV plasmids were then transfected into Huh7 cells, respectively. WT pHBV1.3 was used as a control. Southern blotting and subsequent densitometry analysis (Figure 2 A) revealed that rtQ267H enhances HBV replication with or without a background of rtM204V or rtL180M/M204V significantly. Mutations rtM204V/I weaken HBV replication, while rtL180M could restore the impaired replication capacity, in accordance with previous report (11). HBsAg and HBeAg levels in culture supernatant at 96 hpt were also measured by ELISA (Figure 2 B).

The results indicated that rtL180M and/or rtM204V/I and/or rtQ267H had no influence on HBsAg and HBeAg expression comparing to that of WT HBV, in spite of the fact that rtM204V and rtM204I led to sI195M and sW196S in S ORF, due to overlapping between S and P gene (20).

4.3. Substitution rtQ267H is involved in Reduced Susceptibility to LMV, Aggravated Existing LMV/LdT Resistance Degree of rtM204V

**Figure 3.**
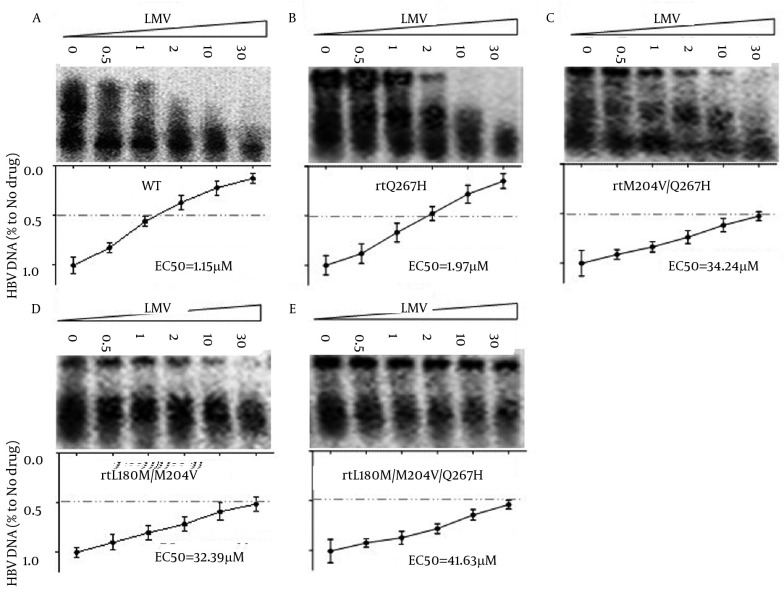
Southern Blot Analysis to Detect the Anti-HBV Effect of LMV towards WT HBV and Different Mutants Huh7 cells were transfected with pHBV1.3 (Figure 3 A), -rtQ267H (Figure 3 B), -rtM204V/Q267H (Figure 3 C), -rtL180M/M204V (Figure 3 D), -rtL180M/M204V/Q267H (Figure 3 E), and then treated with LMV at the indicated concentrations. Encapsulated HBV DNA was extracted and analyzed by Southern blot (upper panel). The relative level of LMV’s anti-HBV effect is shown as a percentage of the control by gray analysis (lower panel).

To determine whether rtQ267H influenced HBV susceptibility to LMV and others NAs, Huh7 cells were transfected with MT or WT HBV plasmids, then exposed to the indicated NA concentrations and the HBV core-associated DNA was analyzed by Southern blot. As shown in Figure 3 A, LMV inhibited the replication of WT HBV in a dose-dependent manner, while the susceptibility of pHBV1.3-rtQ267H, -rtM204V/Q267H, -rtL180M/M204V and -rtL180M/M204V/Q267H to LMV and LdT was greatly reduced

The relative level of LMV’s anti-HBV effect is shown as a percentage of the control by gray analysis (lower panel), and the EC50 of pHBV1.3-WT, -rtQ267H, -rtM204V/Q267H, -rtL180M/M204V and -rtL180M/M204V/Q267H HBV to LMV was calculated by gray analysis were 1.15, 1.97, 34.24, 32.39, 41.63 μM, respectively. These values were approximate to the following values calculated by qRT-PCR analysis. It follows that rtQ267H could not only reduce the anti-HBV effect of LMV, but also enhance HBV replication which was attenuated by LMV resistant mutation, such as rtM204V/I.

**Figure 4.**
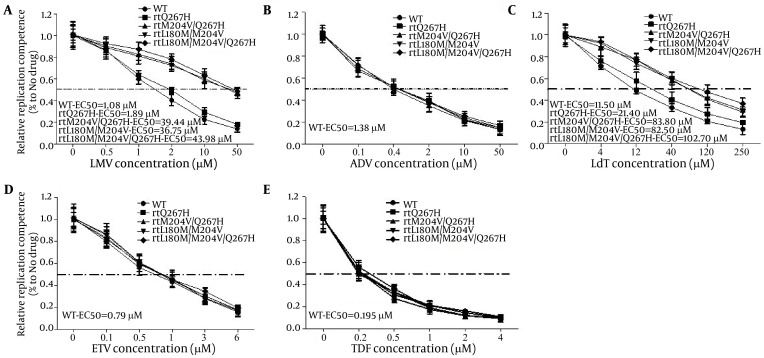
qRT-PCR Was Performed to Detect the Anti-HBV Effects of Five Kinds of Nas Huh7 cells were transfected with pHBV1.3, -rtQ267H, -rtM204V/Q267H, -rtL180M/M204V, -rtL180M/M204V/Q267H, and then treated with LMV at the indicated concentrations. Encapsulated HBV DNA was extracted and analyzed by qRT-PCR to evaluate the anti-HBV effects of LMV (A), ADV (B), LdT (C), ETV (D), and TDF (E). The results shown were calculated as means ± 2 SDs (n = 3).

To calculate how much influence MTs HBV gave rise to, qRT-PCR targeted to HBV RC DNA was performed. As shown in Figure 4, the half maximal effective concentration (EC50) of pHBV1.3-WT, -rtQ267H, -rtQ267H/M204V, -rtL180M/M204V and -rtL180M/M204V/Q267H to LMV were about 1.08, 1.89, 39.44, 36.75. and 43.98 μM, respectively, as calculated by qRT-PCR, which was much higher than that of WT (1.08 μM). Their resistance indexes (the EC50 value of MT divided by that of WT) to LMV were about 1.75, 36.52, 34.02, and 40.72, respectively (Figure 4 A).

For LdT, similar to LMV, the EC50 of pHBV1.3-WT, -rtQ267H, -rtQ267H/M204V, -rtL180M/M204V and -rtL180M/M204V/Q267H to LdT were about 11.50, 21.40, 83.80, 82.50, and 102.70 μM, respectively. And their resistance indexes were about 1.86, 7.65, 7.17, and 8.94, respectively (Figure 4 C).

### 4.4. Substitution rtQ267H Maintained Normal Susceptibility to Other NAs, Except for LMV and LdT

rtQ267H, rtQ267H/M204V, rtL180M/M204V, and rtL180M/M204V/Q267H substitutions reduced the anti-HBV effect of LMV and LdT. We further analyzed their susceptibility to other NAs. Huh7 cells were transfected with WT pHBV1.3, pHBV-rtQ267H, -rtQ267H/M204V, -rtL180M/M204V and -rtL180M/M204V/Q267H, then treated with indicated concentrations of ADV, ETV and TDF. qRT-PCR was used to measure the HBV RC-DNA copy number and the EC50 of WT and MT HBV to ADV (Fig 4B), ETV (Figure 4 D) and TDF (Figure 4 E). Their EC50 values to ADV, ETV and TDF were all close to that of WT (1.38, 0.79 and 0.195 μM, respectively). The results indicated that rtQ267H, rtQ267H/M204V, rtL180M/M204V, and rtL180M/M204V/Q267H decreased the susceptibility of HBV to LMV and LdT, but they were still susceptible to ADV, ETV, and TDF in vitro.

## 5. Discussion

Chronic infection with HBV can result in cirrhosis and hepatocellular carcinoma, giving rise to a worldwide health problem (21), but it could be effectively improved by NAs (22), which were widely used to treat acquired immune deficiency syndrome (Aids) patients originally (11, 23, 24). Unfortunately, drug resistance caused by mutations in HBV-RT severely impaired HBV therapy, led to virological breakthrough, hepatitis aggravation, and so on (8, 24). In spite of approving anti-HBV effect, popular price and good patient's compliance, LMV resistance increases progressively during the course of treatment, LMVr is taken place in about 14%–32% CHB patients each year after the treatment initiation, and more than 80% are resistant after four years treatment. The rate of emergence of LdT resistant HBV is lower than LMV. Due to infection with drug-resistant viral strains, patients would experience virologic breakthrough or partial response to their primary therapy. Treatment with a second NA is necessary.

Generally speaking, it is extremely important to find resistance mutations and adjust therapeutic schedule timely. In our study, CHB patients were all accepted LMV and/or ADV anti-HBV therapy, LMV and ADV-associated mutations were focused respectively. ADV-associated mutations were reported in our previous paper (25), while the present study focused on LMV-related mutations. Through sequence analysis of the samples from patients’ serum, a series of mutations were found, except for existing reported LMVr mutations only rtQ267H was newly found closely related to LMV usage in all patients. In the following step, rtQ267H was introduced into WT or MT replication-competent plasmid to demonstrate that it was not only responsible for enhancing HBV replication, but also aggravating LMV resistance degree of known LMV-resistant mutants in vitro, such as rtL180M/M204V (26). Our study provided a common and effective method to know what mutations took place, which drug was invalid, and which one should be allowed for successive use. Although the emergence of rtQ267H may be more serious for CHB patients with LMVr/LdTr, fortunately ADV, ETV and TDF showed comparative antiviral activity against LMVr/LdTr HBV mutants and WT HBV in vitro.

The efficacy and safety profile of NAs in HBV infection have already been studied in detail worldwide. Four kinds of nucleic acids (dA/T/G/CTP) are indispensable material for DNA synthesis. NAs are very similar with dA/T/G/CTP, so that they could mimic physiological nucleosides for uptake and metamorphosis, be incorporated into newly synthesized viral DNA giving rise to synthesis inhibition and termination. Substitution rtM204V/I significantly changes the three-dimensional structure of HBV-RT, thus it would be difficult for LMV and LdT to enter the catalytic pocket or separate from the catalytic pocket when this mutation take place. Furthermore, physiological nucleosides are also prevented from entering catalytic pocket of the mutated HBV-RT and then the HBV replication capacity would be influenced, as shown in Figure 2 A. Due to selection advantage under high selection pressure of LMV or LdT, HBV virions with rtM204V/I, especially together with rtL180M and/or rtL80I and/or rtV173L at the same time, would be selected as dominant strains. Substitutions rtL180M /rtL80I/rtV173L which can restore the impaired HBV replication ability, are called adaptive mutations of HBV to LMV. In our study, besides these three mutations, another adaptive mutation rtQ267H was found, not only restored the HBV replication, but also exacerbated LMVr and LdTr. The result enriches existing spectrum of LMVr/LdTr mutations.

Based on the homology model of HBV-RT built in our previous study (11), the distances of a given aa residue in HBV polymerase to a given NA substrate could be estimated by comparison with HIV RT. The azimuthal variation between DNA template, primer oligonucleotides, and WT/MT HBV-RT could also be simulated (11). In further research, we plan to bring rtQ267H to the model of HBV-RT to study the association between LMV/LdT and rtQ267H. Hydrodynamically injected mice HBV model was used widely in our previous research to analyze the drug-resistant phenotype of HBV; we would explore the susceptibility of WT/LMVr HBV carrying rtQ267H to NAs with replication-competent pAAV-HBV1.3.
